# CAT correlates positively with respiratory rate and is a significant predictor of the impact of COPD on daily life of patients: a cross sectional study

**DOI:** 10.1186/2049-6958-9-47

**Published:** 2014-09-09

**Authors:** Cíntia Becker, Janaína Schäfer, Lisiane L Carvalho, Isabel P Vitiello, Andréa LG da Silva

**Affiliations:** Physiotherapist at CCGS` Hospital Physiotherapy Service, Santa Cruz, Brazil; Scientific Initiation Fellow by University of Santa Cruz do Sul – UNISC, Santa Cruz, Brazil; Physiotherapist and Master Student by Research Group “Health Rehabilitation and its Interfaces” – UNISC, Santa Cruz, Brazil; Professor by Research Group “Health Rehabilitation and its Interfaces” – UNISC, Santa Cruz, Brazil; Professor and coordinator of Research Group “Health Rehabilitation and its Interfaces” – UNISC, 1780/15 Bairro Santo Inácio, Santa Cruz do Sul, RS 96820-180 Brazil

**Keywords:** CAT, COPD, Dyspnea, Rehabilitation

## Abstract

**Background:**

The pathophysiological changes of COPD tend to worsen with progression, triggering limiting symptoms and implying the decrease in the activities of daily living and quality of life. The COPD Assessment Test (CAT) is a questionnaire designed to measure the impact of COPD on the health status. The aim of this study was to evaluate the impact of the disease through the CAT in a Brazilian sample of COPD patients and to correlate symptoms at rest with the CAT score in these patients.

**Methods:**

Study of cases with COPD patients was conducted by pulmonary rehabilitation program (RP). Respiratory rate (RR) and symptoms (dyspnea by Modified Borg Scale Dyspnea Index; symptoms by CAT) were analyzed at the beginning of the RP.

**Results:**

The study analyzed 28 COPD patients, both genders, age 65.93 ± 7.84 years and many patients ranging from severe and very severe disease. The majority of patients were rated by CAT with low impact-disease (n = 13/46, 4%);medium (n = 11/39, 3%) and the high impact-diseases were observed in a few subjects (n = 4/14.3%). The difference between all CAT scores was significant, p = 0.000. There was a positive correlation between respiratory rate and CAT scores impact-level (r = 0.585, p = 0.001). The results obtained by the Borg Scale revealed a high presence of symptoms in these COPD patients but no association with CAT.

**Conclusion:**

The CAT is a sensitive tool to assess the current health status of COPD patients, and in Southern Brazil it is positively correlated with respiratory rate.

## Background

Chronic Obstructive Pulmonary Disease (COPD) is one of the leading causes of mortality worldwide and it has been estimated that 80 million people have the disease classified as moderate to severe [1]. COPD is characterized by progressive and irreversible limitation of airflow and one of the main objectives of its treatment is to warrant that the patient's health is controlled. However, despite the availability of clinical guidelines for disease control, a significant number of patients do not achieve the level of success that can be attained with treatment [2, 3].

The physiopathological alterations in COPD tend to worsen with disease progression and to trigger limiting symptoms, which result in a decrease in activities of daily living and quality of life [2]. Due to this process, patients enter a vicious circle, in which they limit their activities to relieve symptoms [1, 3]. The aims of COPD treatment include preventing disease progression, relieving symptoms, improving exercise tolerance and health status, preventing and treating complications and exacerbations, reducing mortality and preventing or minimizing side effects throughout the treatment program [1, 4]. The COPD Assessment Test (CAT) aims to help patient and health care to measure the impact of COPD on the well-being and daily life of the individual, facilitating the improvement of disease management and obtaining maximum benefits from the treatment [2].

The CAT is a questionnaire developed to provide a measure of the impact of COPD on the health status of the patient, used to monitor disease progression and assist in the decision making on the treatment of COPD patients [4]. CAT identifies the key points that the physician or physiotherapist can explore during consultation. It also provides a reliable measure of disease severity, regardless of the language spoken. This should ensure that it is relevant for an international population of COPD patients and applicable at worldwide level [5].

The high incidence of COPD in the South Brazil and the consequent higher rate of disease-related mortality in this region justify performing a study to evaluate the implementation of CAT. Although pulmonary rehabilitation and COPD are well-known and discussed topics among physiotherapists, little is known about this new questionnaire as means of assessment to assist in COPD management. It is believed that the implementation of this tool as means of assessing disease impact can result in large benefits in the health management of these patients. Therefore, the aim of this study was to evaluate the impact of the disease through the CAT in a Brazilian sample of patients with COPD, and to correlate symptoms at rest with the CAT score in these patients.

## Methods

This investigation was carried out in the city of Santa Cruz do Sul, state of Rio Grande do Sul, Brazil, in the settings of pulmonary rehabilitation of Hospital Santa Cruz (HSC). The study was approved by the Ethics Committee in Research of Santa Cruz do Sul University, protocol number 3016/11.

It is characterized as a cross-sectional study. It included 28 patients with a clinical diagnosis of COPD and controlled disease that attended a pulmonary rehabilitation program. We excluded individuals with orthopedic, disabling central nervous system or peripheral disorders that were unable to participate in the pulmonary rehabilitation program, as well as other associated pulmonary diseases.

For the evaluation of symptoms and impact on individuals’ lifestyle, the COPD Assessment Test (CAT) questionnaire was applied. The CAT obtains reliable and valid data on the impact of COPD on health status, such as information on daily symptoms, activity limitations and other disease manifestations [2]. The questionnaire contains 8 simple items that describe the scenario from the best to the worst, which are added resulting in a final single score (minimum 0, maximum 40). If the score is less than 10, the symptoms are considered mild; from 10 to 20, moderate; 21 to 30, severe; and more than 30, very severe [5].

Once the individuals came to pulmonary rehabilitation to perform the activities according to the program schedule, the data were collected. Thus, after 5 minutes of rest, data were collected on respiratory rate, dyspnea at rest by Modified Borg Scale Dyspnea Index and then the CAT was applied. Demographic data (age), anthropometrics (BMI) and lung function were compiled from the existing database.

### Statistical analysis

After collection, the data were analyzed using the Statistical Package SPSS, release 18.0 and p ≤ 0.05 was considered statistically significant. Data were expressed as mean ± standard deviation. For multiple comparisons between groups, one-way analysis of variance (ANOVA) was used with Tukey's *post hoc* test. The associations were performed by Spearman’s correlation coefficient.

## Results

The study included 28 COPD patients whose clinical characteristics, smoking status and home oxygen therapy data are described in Table 1.

**Table 1:** **Clinical characteristics of COPD patients**

Characteristics	COPD (n = 28)
Age (years)	65.93 ± 7.84
BMI (kg/m^2^)	24.10 ± 5.78
Gender (n)	17
Oxygen therapy (yes)^†^	05
Current smoker (n)	03
Ex- smoker (n)	24
Never smoker (n)	01
FEV_1_ (% predicted)	37.25 ± 18.67
FVC (% predicted)	59.90 ± 19.04
FEV_1_/FVC (% predicted)	60.50 ± 16.89
CODP stage*	
Mild and moderate (n)	08
Severe and very severe (n)	20

Data are presented as mean ± SD; ^†^Overnight oxygen therapy; ^*^Mild/Moderate/Severe/Very Severe (GOLD, 2014).

BMI,Body Mass Index; n, number sample; FEV_1_, forced expiratory volume in one second; FVC, forced vital capacity.

**Figure 1:**
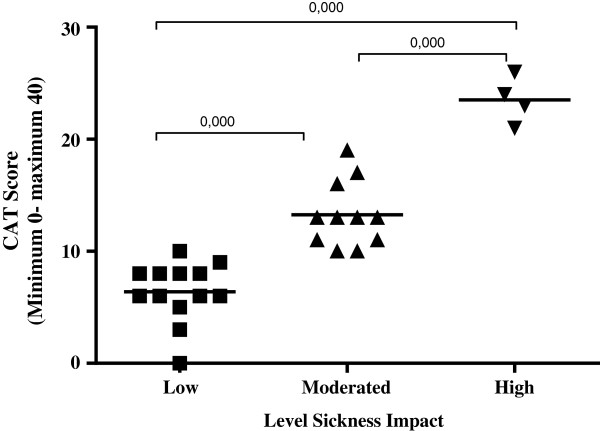
**Level impact of COPD as to** **CAT score.** Data are presented as mean ± standard deviation. Statistical analysis was performed by one-way ANOVA with Tukey's multiple comparison test.

**Figure 2:**
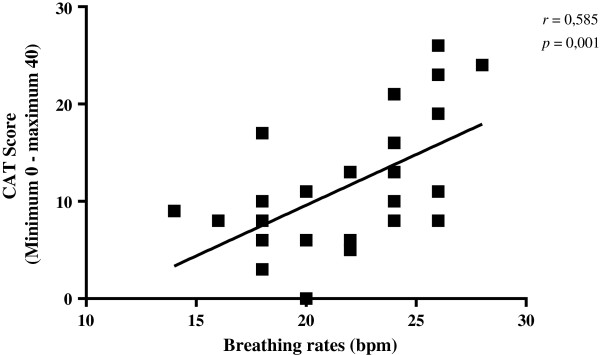
**Correlation between CAT score and respiratory frequency in patients with COPD.** bpm, breaths per minute. Statistical analysis was performed by Spearman`s correlation coefficient.

Regarding lung function, according to the percent (%) of predicted FEV_1_ values [1], COPD patients were classified as stage mild to moderate disease (GOLD I and II) and severe to very severe disease (GOLD III and IV) (Table 1).The Figure 1 shows the CAT classification regarding the impact of the disease on patients’ daily lives in the data collection day. It was observed that most patients were classified as having low (n = 13/46.4%) and moderate impact (n = 11/39.3%), whereas high impact was observed in a few individuals (n = 4/14.3%) and no patient was classified as having very high impact. It is important to emphasize that, although this classification shows a range previously defined in the literature, the difference observed between the means of this study was statistically significant.There was a positive and moderate correlation between respiratory rate and CAT score/level of impact, (i.e., the higher the respiratory rate, the higher the final score at the questionnaire), indicating a high impact of COPD on the subject’s health status (Figure 2).

No significant difference was observed in the CAT score stratified by grouping of COPD, according to disease staging in two main groups: Mild Disease (Mild + Moderate COPD stage/CAT = 10.56 ± 7.95) and Severe Disease (Severe + Very Severe COPD stage/CAT = 12.00 ± 5.85, p = 0.592). The dyspnea degree assessment obtained using the Modified Borg Scale is shown in Table 2.

**Table 2:** **Classification of dyspnea by Modified- Borg Scale of breathlessness**

Classification of dyspnea	COPD (n = 28)
Mild (n)	07
Moderate (n)	12
Intense (n)	08
Very intense (n)	01
Data are presented as mean ± standard deviation.	

It can be observed that the results obtained using the Modified Borg Scale adapted for dyspnea showed the presence of significant symptoms in these subjects. However, no correlation was observed between the Borg scale and the CAT in this study.

## Discussion

The present study investigated the implementation of the CAT in a pulmonary rehabilitation program for COPD patients, in the Southern region of Brazil, with a predominance of Caucasian individuals of European descent. According to the results shown in Table 1, in this study most subjects were characterized as having COPD in the severe stage. Although spirometry results have a direct association with respiratory functions, any increase in spirometric results does not necessarily indicate a possible increase in quality of life. FEV_1_ is a useful indicator of airflow obstruction, but has no value for the assessment of dynamic hyperinflation. Consequently, the measurement of quality of life together with the spirometry results can be useful for the treatment of patients with COPD [6].Despite the deterioration in lung function observed by spirometry test, the level of disease impact assessed by CAT ranged from low to moderate (Figure 1). It is noteworthy that no significant difference was observed in the CAT score stratified by grouping of COPD, as mild and severe stage.

In addition to the routine clinical evaluation, a critical step in disease management is to obtain reliable and valid data on the impact of COPD on the individual’s health status. The CAT must also be able to identify specific areas of higher severity to be used as the focal point for evaluation of goals, thereby improving the care process and outcomes [2]. Patients with more severe airway obstruction and frequent exacerbations have higher scores on CAT than patients with moderate COPD [2, 7].

The CAT includes items related to cough, sputum production, chest tightness, exercise and activity capacity, trust, quality of sleep and energy levels. It is an appropriate questionnaire for routine clinical use that shows reliability and validity in patients with stable and exacerbated COPD [7]. It was developed to provide a measure of the impact of COPD on the health status of an individual in order to help address these conditions and improve disease management [5].

Another aspect to be highlighted is the fact that the study sample consists of COPD patients undergoing treatment in a pulmonary rehabilitation program. These observations suggest that CAT is a sensitive tool to health alterations, even in stable individuals undergoing pulmonary rehabilitation because the exercise has a significant impact on the psychological responses to reduce psychological distress and improve positive well-being [8]. It was developed to meet the need of performing a reliable and practical measurement of health status. It is a simple tool, and when used as part of routine clinical practice, it evaluates the impact of symptoms more easily than the more complex measures that are currently used [7, 9].

The respiratory rate, considered a parameter of cardiorespiratory assessment and monitoring [10], positively correlated with CAT (Figure 2).

During patient follow up, it is important to highlight the impact of clinical practice on symptom control, mainly difficulty in breathing, and this has a clear effect on patient quality of life, whereas other more objective parameters (such as spirometry data) are not always so directly related to this aspect. For this reason, the management expressed in COPD guidelines is not appropriate for the symptoms of individuals [5, 10]. Specific questionnaires related to COPD and adapted to our environment are useful tools in assessing the effectiveness of interventions in these patients [4].

Even though the CAT has been validated for the Brazilian population and is applicable to international populations, there was some difficulty in interpreting the results considering the clinical characteristics and pulmonary function parameters of the study subjects. It is worth mentioning that some factors often contribute to the inadequate management of COPD and one of them is the fact that patients understate the severity of their conditions [5]. This statement is consistent with the results of other studies, where the majority of COPD patients, despite the severe staging of the disease and presence of significant symptoms (Table 2), showed a low impact of the disease and none showed very high impact (Figure 1).

The management of most chronic diseases is also characterized by patients’ extensive responsibility [11], as they should understand and approve the new practices and responsibilities [12].

Given this context, it is vital to remember that, in addition to the pulmonary alterations resulting from the disease, COPD is associated with comorbidities, such as anxiety, depression and atherosclerotic diseases, as well as cognitive dysfunctions involving attention deficit, perception, learning and memory. The cognitive dysfunction has a specific pattern in COPD, being considered mild in non-hypoxemic COPD and high in hypoxemic patients [13]. Moreover, the most affected cognitive domains found in patients with COPD are: memory, attention, speech, coordination and learning skills [14].

### Limitation of the study

This study has limitations like the lack of dyspnea- MRC score and modified Borg scale to evaluate fatigue sensation of the COPD patients.

## Conclusion

Considering the above, it is believed that the CAT is a sensitive tool to assess the current health status of COPD patients, in the region of Southern Brazil, and it is positively correlated with respiratory rate. Specific questionnaires related to COPD and adapted to our environment are useful tools to assess the effectiveness of interventions in these patients.
